# Quaternions as a solution to determining the angular kinematics of human movement

**DOI:** 10.1186/s42490-020-00039-z

**Published:** 2020-03-23

**Authors:** John H. Challis

**Affiliations:** Biomechanics Laboratory, Pennsylvania State University, University Park, PA 16802 USA

**Keywords:** Quaternion, Orientation, Singularity, Averaging, Interpolation

## Abstract

The three-dimensional description of rigid body kinematics is a key step in many studies in biomechanics. There are several options for describing rigid body orientation including Cardan angles, Euler angles, and quaternions; the utility of quaternions will be reviewed and elaborated.

The orientation of a rigid body or a joint between rigid bodies can be described by a quaternion which consists of four variables compared with Cardan or Euler angles (which require three variables). A quaternion, *q* = (*q*_0_, *q*_1_, *q*_2_, *q*_3_), can be considered a rotation (Ω = 2 cos^−1^(*q*_0_)), about an axis defined by a unit direction vector $\left({q}_1/\sin \left(\frac{\Omega}{2}\right),{q}_2/\sin \left(\frac{\Omega}{2}\right),{q}_3/\sin \left(\frac{\Omega}{2}\right)\right)$. The quaternion, compared with Cardan and Euler angles, does not suffer from singularities or Codman’s paradox. Three-dimensional angular kinematics are defined on the surface of a unit hypersphere which means numerical procedures for orientation averaging and interpolation must take account of the shape of this surface rather than assuming that Euclidean geometry based procedures are appropriate. Numerical simulations demonstrate the utility of quaternions for averaging three-dimensional orientations. In addition the use of quaternions for the interpolation of three-dimensional orientations, and for determining three-dimensional orientation derivatives is reviewed.

The unambiguous nature of defining rigid body orientation in three-dimensions using a quaternion, and its simple averaging and interpolation gives it great utility for the kinematic analysis of human movement.

## Background

The discovery of quaternions is generally attributed to William Rowan Hamilton (1805–1865) who had a sudden insight when walking with his wife on October 16, 1843. He was so excited by this insight, generalizing complex numbers into three-dimensions, that he carved a key formula and the date into the Broome Bridge in Dublin. He wrote,

$$ {i}^2={j}^2={k}^2= ijk=-1 $$

The quaternion was therefore,

$$ q={q}_0+{q}_1i+{q}_2j+{q}_3k $$

Where *q*_0_, *q*_1_, *q*_2_, and *q*_3_ are all real, and the imaginary components (*i, j, k*) are the fundamental quaternion units having the rules for multiplication inscribed on Broome Bridge. The name quaternion comes from the Latin *quaternio*, meaning a group of four. The term had been previously used to refer to a group of four soldiers by Milton in *Paradise Lost (1663)*, and by Scott in *The Waverly Novels* (1832) to refer to a word with four syllables.

Although others had envisaged quaternions before Hamilton, for example Olinde Rodrigues [16] and Leonhard Euler [12], it was Hamilton who first started to formalize their algebra. William Thomson (1824–1907) an Irish mathematical physicist claimed that,

“*Quaternions came from Hamilton after his really good work had been done, and though beautifully ingenious, have been an unmixed evil to those who have touched them in any way*.” [23]

Not all scientists of the time were scornful of quaternions (e.g., [6]). In the last century the development of computers, the introduction of computer graphics, and the automation of the capture of rigid body motion have revealed the full utility of the quaternions in modern biomechanics.

The three-dimensional description of rigid body kinematics is a key step in many studies in biomechanics. Once measured, kinematic data may require numerical procedures such as interpolation, averaging, and differentiations. For three-dimensional linear kinematic data these procedures are relatively straightforward as linear kinematics are defined in three-dimensional Euclidean space. In contrast, three-dimensional angular kinematics are defined on the surface of a unit hypersphere [14], as a consequence different numerical procedures are required for operations such as interpolation and averaging. The use of quaternions helps simplify some of these numerical procedures, and provide some advantages over other methods of describing three-dimensional angular kinematics such as Cardan and Euler angles. Here the utility of quaternions will be presented for: representations of rigid body orientation, determining three-dimensional orientation, avoiding singularities, averaging three-dimensional orientation, interpolating three-dimensional orientations, and for determining three-dimensional orientations derivatives. Where appropriate the performance of quaternions will be juxtaposed with that of Cardan and Euler angles. This review commences with a presentation of the general properties of quaternions.

## Quaternions

There are three common ways of presenting quaternions. The first is as a complex number with three imaginary parts,

1 $$ q={q}_0+{q}_1i+{q}_2j+{q}_3k $$

Where *q*_0_, *q*_1_, *q*_2_, and *q*_3_ are all real, and *i, j, k* are the imaginary components. The second is 7as a vector with four components,

2 $$ q=\left({q}_0,{q}_1,{q}_2,{q}_3\right) $$

This representation is the four-tuple form of the quaternion. Finally, the quaternion can be represented as a scalar (*q*_0_) and a three element vector ($\underset{\_}{q}=\left(\ {q}_1,{q}_2,{q}_3\right)$),

3 $$ q=\left(\ {q}_0,\underset{\_}{q}\right) $$

**Fig. 1:**
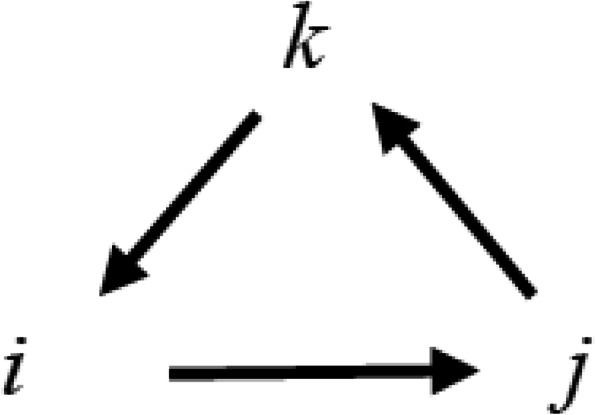
Products of imaginary numbers comprising a quaternion. Given any starting point, moving in a counter-clockwise direction (with the arrows) gives the results of the products of the imaginary numbers (e.g., *k.i = j*). If the motion is clockwise then the product is negative (e.g., *j.i = −kj*)

The products of the imaginary numbers can be described using the following Figure (Fig. 1).

There are a number of types of quaternions, given that the norm of a quaternion is,

4 $$ \mid q\mid =\sqrt{q_0^2+{q}_1^2+{q}_2^2+{q}_3^2} $$

the primary ones are,

Pure quaternion *q* = (0, *q*_1_, *q*_2,_*q*_3_)

Identity quaternion *q* = (1, 0, 0, 0)

Conjugate quaternion $$ \overline{q}=\left({q}_0,-{q}_1,-{q}_{2,}-{q}_3\right) $$

Quaternion Inverse $$ {q}^{-1}=\frac{\overline{q}}{{\left|q\right|}^2} $$

Unit Quaternion *q* = (*q*_0_, *q*_1_, *q*_2,_*q*_3_)

(where) ∣*q* ∣  = 1

The norm for the unit quaternion is equal to one, its inverse is therefore simply its conjugate. For describing rotations in three-dimensions unit quaternions are used, their intrinsic properties confering a number of advantages.

## Representations of rigid body orientation

If a rigid body in three-dimensions undergoes translation and rotation then the new pose (position and orientation) of any point on that body can be described by,

5 $$ y= Rx+\underset{\_}{v} $$

Where *y* are points measured in pose 2, *R* is a *3* × *3 a*ttitude matrix, *x* are points measured in pose 1, and $\underset{\_}{v}$ is a *3* × *1* vector describing the translation from one pose to the other. The attitude matrix belongs to the special-orthogonal group of order three, *R* ∈ *SO*(3). As a consequence of being in this group the inverse of the attitude matrix also belongs to the special-orthogonal group, as does the product of any matrices in this group,

$$ {R}^T={R}^{-1}\kern0.5em R{R}^T={R}^TR=I\kern0.5em \det (R)=1 $$

The attitude matrix consists of nine direction cosines, but these elements do not convey the nature of three-dimensional rotations.

Derived from the work of Cayley [3] there is a relationship between the attitude matrix and a skew-symmetric matrix *P*,

6 $$ R=\left(I-P\right){\left(I+P\right)}^{-1} $$

Where *I* is the identity matrix, and *P* is a skew-symmetric matrix which has the following format,

7 $$ P\left\{p\right\}=\left[\begin{array}{ccc}0& -{p}_3& {p}_2\\ {}{p}_3& 0& -{p}_1\\ {}-{p}_2& {p}_1& 0\end{array}\right] $$

This analysis suggests that as *P* only has three unique elements; in theory the attitude matrix can be described by three elements only. The most common of these are the Cardan and Euler angles (e.g., [26]). Both of these angle conventions can be described as an ordered sequence of rotations about three coordinate axes. For the Cardan angles a sequence might be rotations about the *X*, *Y*, and *Z* axes respectively,

8 $$ {R}_{XYZ}={R}_Z\left(\gamma \right){R}_Y\left(\beta \right){R}_X\left(\alpha \right) $$

Where *α, β*, and *γ* are angles of rotation about the *X Y*, and *Z* axes respectively. For the Euler angles a sequence might be rotations about the *Z*, *Y*, and *Z* axes respectively,

9 $$ {R}_{ZXZ}={R}_Z\left(\gamma \right){R}_X\left(\beta \right){R}_Z\left(\alpha \right) $$

For this convention, the terminal rotations use the same axis, but in theory that axis has already been rotated by the middle rotation in the sequence so is in a different orientation for the second rotation about the axis. For both Cardan and Euler angles each can use six different permutations of axes.

For the *Z, X*, *Z* Euler sequence the attitude matrix can be expressed in terms of the three Euler angles (*γ*, *β*, *α*),

10 $$ {R}_{ZXZ}=\left[\begin{array}{ccc}c\left(\alpha \right)c\left(\beta \right)c\left(\gamma \right)-s\left(\alpha \right)s\left(\gamma \right)& -c\left(\alpha \right)c\left(\beta \right)s\left(\gamma \right)-s\left(\alpha \right)\mathit{\cos}\left(\gamma \right)& c\left(\alpha \right)s\left(\beta \right)\\ {}s\left(\alpha \right)c\left(\beta \right)c\left(\gamma \right)+c\left(\alpha \right)s\left(\gamma \right)& -s\left(\alpha \right)c\left(\beta \right)s\left(\gamma \right)+c\left(\alpha \right)c\left(\gamma \right)& s\left(\alpha \right)s\left(\beta \right)\\ {}-s\left(\beta \right)\mathrm{c}\left(\gamma \right)& s\left(\beta \right)s\left(\gamma \right)& c\left(\beta \right)\end{array}\right] $$

Note that *cos*(*α*) is represented by represented by *c*(*α*), and *sin*(*α*) by *s*(*α*) and similarly for the other angles. Inspection of the matrix reveals how the individual angles can be extracted from the matrix,

11 $$ \mathit{\cos}\left(\beta \right)={r}_{3,3} $$

12 $$ \mathit{\sin}\left(\alpha \right)=\frac{r_{2,3}}{\mathit{\sin}\left(\beta \right)} $$

13 $$ \mathit{\sin}\left(\gamma \right)=\frac{r_{1,3}}{\mathit{\sin}\left(\beta \right)} $$

If there is only rotation about one axis then it is relatively easy to visualize the change in orientation described by a set of Euler or Cardan angles, but it is harder when there is motion about two or three of the axes.

The change of rigid body orientation described by quaternions adds one more variable compared with Cardan or Euler angles (from three to four). A quaternion, *q* = (*q*_0_, *q*_1_, *q*_2_, *q*_3_), can be considered a rotation of angle Ω, about an axis defined by the unit direction vector *e*, where,

14 $$ {q}_0=\pm \cos \frac{\varOmega }{2} $$

and

15 $$ \left[\begin{array}{c}{q}_1\\ {}{q}_2\\ {}{q}_3\end{array}\right]=\pm \underset{\_}{e}\mathit{\sin}\frac{\varOmega }{2} $$

Where 0 ≤ *Ω* ≤ *π*. Therefore a quaternion can be directly visualized as a directed line in space about which there is a rotation. For example, see Fig. . 2, if a point *r*_*0*_ is transformed by a rotation matrix to point *r*_*1*_, then then this transformation can be visualized as a rotation (Ω) about a line (*e*).

**Fig. 2:**
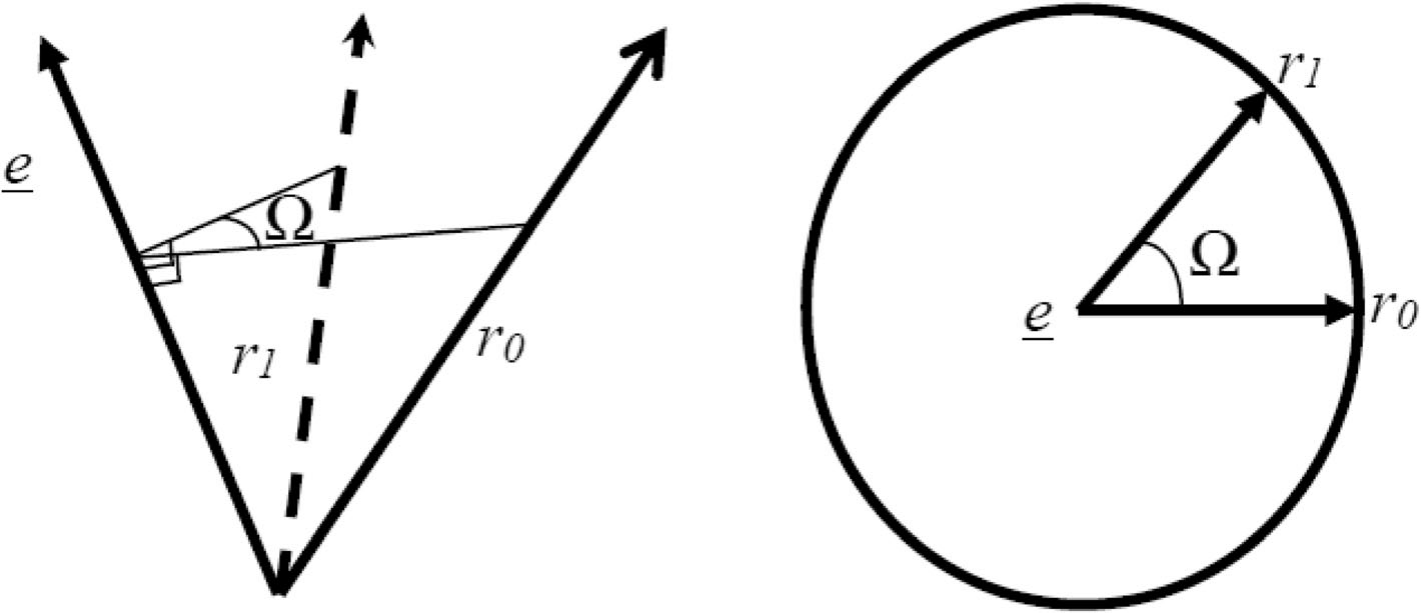
The transformation of a point *r*_0_ by a rotation of Ω about a line *e,* to point *r*_1_. The left image shows the general representation of the transformation, and the right image shows a view in a plane normal to the axis of rotation

Therefore the change in the orientation of a rigid body can be visualized from its quaternion. The problem with characterizing a rotation using Cardan or Euler angles is that the user must define the axis sequence with each sequence corresponding to a different set angles for describing the same rigid body attitude, adding to the problems with this visualization (see Table 1). There is no such ambiguity in quaternions.

**Table 1:** The influence of different angle sequences on the resulting amounts of rotations about each axis for six different Cardanic angle sequences

Angle Sequence	Rotation about Specified Axis (Degrees)		
	***X***	***Y***	***Z***
X-Y-Z	60.00	5.00	−10.00
X-Z-Y	59.12	5.08	−9.96
Y-Z-X	59.63	11.15	−0.72
Y-X-Z	59.62	9.93	−1.42
Z-X-Y	57.70	21.21	−18.89
Z-Y-X	59.49	11.15	−0.73

From Eqs. 8 and 9 it can be seen that angles as defined by Cardan or Euler angles can be combined by taking the product of the matrices describing the rotations to be combined. In a similar fashion if the rotations described by two quaternions (*q* and *r*) are to be combined the quaternion product must be computed,

16 $$ qr=\left[\begin{array}{cccc}{q}_0& -{q}_1& -{q}_2& -{q}_3\\ {}{q}_1& {q}_0& -{q}_3& {q}_2\\ {}{q}_2& {q}_3& {q}_0& -{q}_1\\ {}{q}_3& -{q}_2& {q}_1& {q}_0\end{array}\right]\left[\begin{array}{c}{r}_0\\ {}{r}_1\\ {}{r}_2\\ {}{r}_3\end{array}\right] $$

Quaternion multiplication is not commutative, therefore,

17 $$ \mathrm{qr}\ne \mathrm{rq} $$

Codman’s paradox was identified by Codman in 1934 when examining the function of the shoulder [7]. With the arm in an initial position it is hanging by the side with the thumb towards the front and the fingers pointing down, first rotate the arm to the horizontal (wing position), then rotate arm to the front (fingers are now pointing straight ahead), then bring the arm back down to the side. In this final position the arm has undergone an axial rotation, therefore the thumb is now pointing to the side (inward). In a Cardanic angle sequence this is explained because rotations about the terminal axes (first and third) produce motion about the middle axis. With a quaternion representation the sequence of rotations can be considered as a rotation about each axis in sequence (represented by *i* then *j* then *k*), which results in a rotation at the end of the sequence because *ijk* =  − 1, one of the basic properties of quaternions identified by Hamilton.

Chasles theorem states that the motion of a rigid body can be considered to be a translation along, and a rotation about a suitable axis in space [5]. This theorem means the description of the motion of a rigid body, or motion of one rigid body relative to another rigid body, can be the motion along and around a helical axis. The helical axes have been useful to describe joint behavior (e.g., [1]). The finite helical axis describes the motion of a rigid body from one position to another, and is frequently used as an approximation to the instantaneous helical axis (e.g., [2]). The finite helical axis is defined by: the angle of rotation (Ω) about the axis, the unit direction vector ($\underset{\_}{\mathrm{e}}$) of the axis, the amount of translation (*u*) along the axis, and the location of a point (*s*) on the helical axis. From Eqs. 3, 14, and 15 the angle is computed from,

18 $$ \Omega =2\ {\mathit{\cos}}^{-1}(q) $$

and the unit direction vector ($\underset{\_}{\mathrm{e}}$),

19 $$ \underset{\_}{\mathrm{e}}=\frac{\boldsymbol{q}}{\left|\boldsymbol{q}\right|} $$

The amount of translation (*u*) along the axis comes from,

20 $$ u={\underset{\_}{e}}^T\underset{\_}{v} $$

Finally the location of a point (*s*) on the axis from,

21 $$ s=\frac{1+\mathit{\cos}\left(\Omega \right)}{2{\mathit{\sin}}^2\left(\Omega \right)}\left(I-{R}^T\right)\underset{\_}{v} $$

Eqs. 18 and 19 show the intrinsic relationship between quaternions and finite helical axes.

## Determining the three-dimensional orientation

Determining three-dimensional orientation of a rigid body requires the computation of the attitude matrix (*R*), this occurs in two scenarios. One is the change in pose measured in one reference frame so the attitude matrix would represent the change in orientation. The other is to map from one reference frame to another, typically inertial and body fixed, so the attitude matrix would represent the orientation of one reference frame relative to another. Given the basic rigid body transformation equation, Eq. 5, a least-squares approach to the problem of determining *R* and $\underset{\_}{v}$ would require the minimizing of,

22 $$ \frac{1}{n}\sum \limits_{i=1}^n{\left(R{x}_i+\underset{\_}{v}-{y}_i\right)}^T\left(R{x}_i+\underset{\_}{v}-{y}_i\right) $$

Where *n* is the number of non-coplanar points measured in both reference frames (*n* ≥ 3), *y*_*i*_ is the i^th^ point measured in pose 2, and *x*_*i*_ is the i^th^ point measured in pose 1. If the data are accurate (and noiseless) the result of this equation would be zero, but in reality this does not occur so *R* and $\underset{\_}{v}$ are selected to make the result as close to zero as possible. The identification of *R* and $\underset{\_}{v}$ was presented by Grace Wahba as a numerical problem to solved [24]. Since Wahba presented the challenge solutions have emerged in many domains including photogrammetry (e.g., [9]), mechanical engineering (e.g., [19]), space craft kinematics (e.g., [21]), computer vision (e.g., [10]), and biomechanics (e.g., [4]).

The singular value decomposition [8], can be used to compute *R* and $\underset{\_}{v}$ given measurements of at least three no-coplanar points [4]. It revolves around the decomposition of the cross-dispersion matrix *C* which can be computed from,

23 $$ C=\frac{1}{n}\sum \limits_{i=1}^n{\left({y}_i-\overline{y}\right)}^T\left({x}_i-\overline{x}\right) $$

Where $\overline{x}$ and $\overline{y}$ are the mean vectors ($\overline{x}=\frac{1}{n}\sum \limits_{i=1}^n{x}_i$, $\overline{y}=\frac{1}{n}\sum \limits_{i=1}^n{y}_i$). The singular value decomposition of *C* is computed,

24 $$ C= UD{V}^T $$

Where *U* is a *3 × 3* orthogonal matrix, consisting of vectors *u*_1_, *u*_2,_*u*_3_*, D* is a *3 × 3* diagonal matrix, whose elements are non-negative real values (the singular values), and *V* is a *3 × 3* orthogonal matrix, consisting of vectors, consisting of vectors *v*_1_, *v*_2_, *v*_3_. Then the attitude matrix, *R*, is computed from,

25 $$ R=U\left[\begin{array}{ccc}1& 0& 0\\ {}0& 1& 0\\ {}0& 0& \det \left(U{V}^T\right)\end{array}\right]{V}^T $$

The vector $\underset{\_}{v}$ can be computed using the mean vectors,

26 $$ \underset{\_}{v}=\overline{y}-R\overline{x} $$

Given a unit quaternion the attitude matrix (*R*) can be computed from,

27 $$ R(q)=\left({q}_0^2-{\underset{\_}{q}}^T\underset{\_}{q}\right)I+2\underset{\_}{q}\ {\underset{\_}{q}}^T-2{q}_0S\left\{\underset{\_}{q}\right\} $$

Where $S\left\{\underset{\_}{q}\right\}$ generates a skew-symmetric matrix from a vector, therefore for vector $\underset{\_}{q}=\left({q}_1,{q}_2,{q}_3\right)$,

28 $$ S\left\{\underset{\_}{q}\right\}=\left[\begin{array}{ccc}0& -{q}_3& {q}_2\\ {}{q}_3& 0& -{q}_1\\ {}-{q}_2& {q}_1& 0\end{array}\right] $$

Which when Eq. 27 is expanded gives,

29 $$ R(q)=\left[\begin{array}{ccc}{q}_0^2+{q}_1^2-{q}_2^2-{q}_3^2& 2\left({q}_1{q}_2-{q}_3{q}_0\right)& 2\left({q}_1{q}_3-{q}_2{q}_0\right)\\ {}2\left({q}_1{q}_2+{q}_3{q}_0\right)& {q}_0^2-{q}_1^2+{q}_2^2-{q}_3^2& 2\left({q}_2{q}_3-{q}_1{q}_0\right)\\ {}2\left({q}_1{q}_3-{q}_2{q}_0\right)& 2\left({q}_2{q}_3+{q}_1{q}_0\right)& {q}_0^2-{q}_1^2-{q}_2^2+{q}_3^2\end{array}\right] $$

Note that the matrix is quadratic relative to the quaternions, and unlike other parameters extracted from the matrix does not contain transcendental functions, for example, Eq. 10. This can be an advantage, for example, when fast computations are required.

Inspection of Eq. 29 gives the following equations for the extraction of the quaternions from the attitude matrix,

30 $$ {q}_0=\pm \sqrt{r_{1,1}+{r}_{2,2}+{r}_{3,3}+1} $$

31 $$ {q}_1=\frac{r_{2,3}-{r}_{3,2}}{4\ {q}_0} $$

32 $$ {q}_2=\frac{r_{3,1}-{r}_{1,3}}{4\ {q}_0} $$

33 $$ {q}_3=\frac{r_{1,2}-{r}_{2,1}}{4\ {q}_0} $$

If the quaternion describes a rotation of *π* radians then then *q*_0_ = 0, therefore the remainder of the components of the quaternion are not defined using Eqs. 31, 32, and 33. If the data used to determine the attitude matrix are noisy then this problem can occur as the rotation approaches *π* radians. Shepperd [18] presented a numerically more robust method of extracting the quaternion from the attitude matrix. The first step is the estimate each of the components of the quaternion from,

34 $$ {q_0}^2=\frac{1}{4}\left(1+{r}_{1,1}+{r}_{2,2}+{r}_{3,3}\right) $$

35 $$ {q_1}^2=\frac{1}{4}\left(1+2{r}_{1,1}-\left({r}_{1,1}+{r}_{2,2}+{r}_{3,3}\right)\right) $$

36 $$ {q_2}^2=\frac{1}{4}\left(1+2{r}_{2,2}-\left({r}_{1,1}+{r}_{2,2}+{r}_{3,3}\right)\right) $$

37 $$ {q_3}^2=\frac{1}{4}\left(1+2{r}_{3,3}-\left({r}_{1,1}+{r}_{2,2}+{r}_{3,3}\right)\right) $$

Whichever of these equations provides the largest square root is used as the basis for computing the remainder of the quaternion components using the appropriate equations from the following,

38 $$ {q}_0{q}_1=\frac{1}{4}\left({r}_{2,3}-{r}_{3,2}\right) $$

39 $$ {q}_0{q}_2=\frac{1}{4}\left({r}_{3,1}-{r}_{1,3}\right) $$

40 $$ {q}_0{q}_3=\frac{1}{4}\left({r}_{1,2}-{r}_{2,1}\right) $$

41 $$ {q}_2{q}_3=\frac{1}{4}\left({r}_{2,3}+{r}_{3,2}\right) $$

42 $$ {q}_3{q}_1=\frac{1}{4}\left({r}_{3,1}+{r}_{1,3}\right) $$

43 $$ {q}_1{q}_2=\frac{1}{4}\left({r}_{1,2}+{r}_{2,1}\right) $$

For example, if Eq.35 gives the highest value for a quaternion component, then *q*_0_ is estimated from Eq. 38, *q*_2_ is estimated from Eq. 43, and *q*_2_ is estimated from Eq. 42.

There are numerical methods for determining the quaternion directly from common points measures in two poses (e.g., [9, 22]), these are still based around minimizing Eq. 22 and therefore give equivalent results.

## Avoiding singularities

The attitude matrix consists of nine elements (3 × 3). As this matrix is orthogonal, this property imposes six constraints on its nine elements, a characteristic of matrices belonging to the special-orthogonal group of order three. The constraints suggest that it is feasible to described rigid body orientation using three parameters. However, the three-parameter representations of *SO*(3) for certain rigid body attitudes are singular.

To illustrate the problem with these singularities consider the Cardanic sequence *X-Y-Z* sequence (angles *α, β*, and *γ*),

44 $$ {R}_{XYZ}=\left[\begin{array}{ccc}c\left(\gamma \right)c\left(\beta \right)& c\left(\gamma \right)s\left(\beta \right)s\left(\alpha \right)-s\left(\gamma \right)c\left(\alpha \right)& c\left(\gamma \right)s\left(\beta \right)c\left(\alpha \right)+s\left(\gamma \right)s\left(\alpha \right)\\ {}s\left(\gamma \right)c\left(\beta \right)& s\left(\gamma \right)s\left(\beta \right)s\left(\alpha \right)+c\left(\gamma \right)c\left(\alpha \right)& s\left(\gamma \right)s\left(\beta \right)c\left(\alpha \right)-c\left(\gamma \right)s\left(\alpha \right)\\ {}-s\left(\beta \right)& c\left(\beta \right)s\left(\alpha \right)& c\left(\beta \right)c\left(\alpha \right)\end{array}\right] $$

If the middle rotation *β* = π/2, then then the matrix in Eq. 44 can be expressed in terms of the two terminal angles,

45 $$ {R}_{XYZ}=\left[\begin{array}{ccc}0& \mathit{\cos}\left(\gamma \right)\mathit{\sin}\left(\alpha \right)-\mathit{\sin}\left(\gamma \right)\mathit{\cos}\left(\alpha \right)& \mathit{\cos}\left(\gamma \right)\mathit{\cos}\left(\alpha \right)+\mathit{\sin}\left(\gamma \right)\mathit{\sin}\left(\alpha \right)\\ {}0& \mathit{\sin}\left(\gamma \right)\mathit{\sin}\left(\alpha \right)+\mathit{\cos}\left(\gamma \right)\mathit{\cos}\left(\alpha \right)& \mathit{\sin}\left(\gamma \right)\mathit{\cos}\left(\alpha \right)-\mathit{\cos}\left(\gamma \right)\mathit{\sin}\left(\alpha \right)\\ {}-1& 0& 0\end{array}\right] $$

This can be simplified to,

46 $$ {R}_{XYZ}=\left[\begin{array}{ccc}0& \mathit{\sin}\left(\alpha -\gamma \right)&\ \mathit{\cos}\left(\alpha -\gamma \right)\\ {}0& \mathit{\cos}\left(\alpha -\gamma \right)& -\mathit{\sin}\left(\alpha -\gamma \right)\\ {}-1& 0& 0\end{array}\right] $$

The matrix illustrates that the rotation depends only on the difference between the two angles (*α* − *γ*), and therefore only has one degree of freedom instead of two. The rotation of *β* = π/2 means motions of angles *α* and *γ* results in rotations about the same axis.

Inspection of Eq. 11, 12, and 13 show for an Euler angle sequence of *α, β*, and *γ* the terminal angles (*α*, *γ*) are undefined for *β* angles of *±nπ (n = 0, 1, 2, …)*, because to compute these two angles require division by *sin*(*β*), which is division by zero for *β* values of *±n*π. Similarly for Cardanic angles the terminal angles are undefined for *β* angles of *±(2n + 1)*$\frac{\pi }{2}$ (*n = 0, 1, 2*, …), see Eq. 44.

These singularities can be visualized by considering gimbal mechanism. A gimbal consists of three concentric rings, with axes through each ring representing a rotation. The two inner rings can be rotated so they completely overlap with one another, consequently the rotation about one axis cannot be separated from rotation about the other, thus there is a gimbal lock. In biomechanics to avoid gimbal lock the sequence of rotations for a Cardan or Euler sequence are selected so the system, for example a joint, never approaches a singularity (e.g., [28]).

Quaternions can be represented by positions on the surface of hypersphere, where its radius is equal to the quaternions norm (Fig. . 3). The quaternion representation means that a rigid bodies orientation can be visualized using two quaternions, (*q*_0_*, q*_1_*, q*_2_*, q*_3_) and (-*q*_0_*, -q*_1_*, -q*_2_*, -q*_3_). To avoid this ambiguity quaternions can be constrained to either the top or bottom hemisphere of the hypersphere. Given this constraint there are no singularities or ambiguities with the quaternion definition of rigid body attitude.

**Fig. 3:**
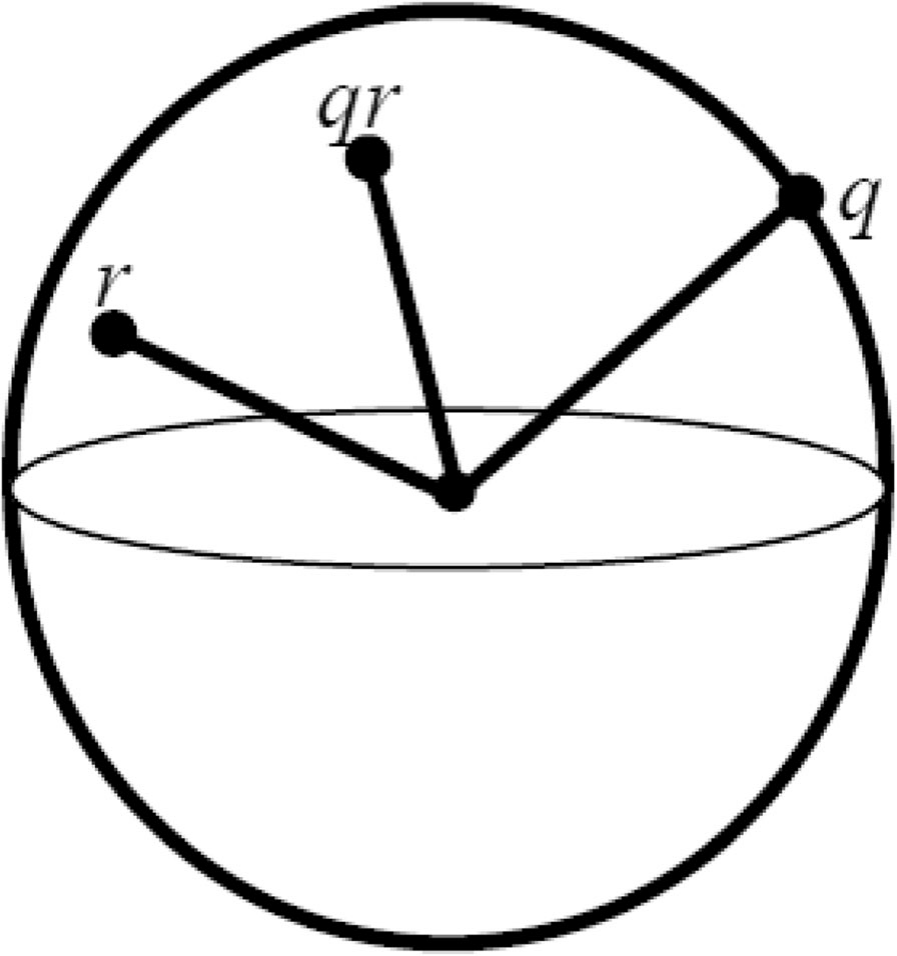
Quaternions represented on a hypersphere, where *q* and *r* are quaternions, and *qr* is the quaternion resulting from their product. Here the quaternions have been constrained to the upper-hemisphere

## Averaging three-dimensional orientations

Three-dimensional angular kinematics are not defined in three-dimensional Euclidean space, unlike linear vectors, but exist on the surface of a non-linear manifold and as a consequence the average orientation is not simply a case of, for example, averaging a set of Cardan angles. Consider the *Y*, *Z*, *X* Cardan sequence with corresponding angles of $\left(\frac{\pi }{2},\frac{\pi }{2},\frac{\pi }{2}\ \right)$, the attitude matrix is,

47 $$ R={R}_X\left(\frac{\pi }{2}\right){R}_Z\left(\frac{\pi }{2}\right){R}_Y\left(\frac{\pi }{2}\right)=\left[\begin{array}{ccc}0& -1& 0\\ {}1&\ 0& 0\\ {}0&\ 0& 1\end{array}\right] $$

If the other Cardan angles to average are $\left(0,0,\frac{\pi }{2}\right)$ the “averaged” set of angles would be $\left(\frac{\pi }{4},\frac{\pi }{4},\frac{\pi }{2}\ \right)$. The error in this analysis is illustrated if the attitude matrix is examined for a *Y*, *Z*, *X* Cardan sequence with corresponding angles of $\left(0,0,\frac{\pi }{2}\right)$,

48 $$ R={R}_X(0){R}_Z(0){R}_Y\left(\frac{\pi }{2}\right)=\left[\begin{array}{ccc}0& -1& 0\\ {}1&\ 0& 0\\ {}0&\ 0& 1\end{array}\right] $$

As three-dimensional rigid body attitude is defined as positions on the surface of hypersphere, the simple averaging of Cardan or Euler angles can produce errors in the average attitude. These errors occur because the averaging of angles is equivalent to taking chords of a circle, but appropriate averaging should take into account the contour of the surface described by the hypersphere. Moakher [15] has demonstrated that the error in averaging the Cardan or Euler angles, for example to average orientations described by *R*_1_ and *R*_2_ is,

49 $$ {d}_E=2\sqrt{2}\left|\mathit{\sin}\frac{\theta }{2}\right| $$

Where $\theta ={\cos}^{-1}\left(\frac{1}{2}\left( tr\left({R}_1^T{R}_2\right)-1\right)\right)$. The equation indicates that if the angular distance (*θ*) across the surface of the hypersphere is too great then the error in the average determined from the Cardan or Euler angles will also be large, quaternions offer a solution to this problem.

The average rigid body attitude can be computed from a sequence of quaternions (*q*_*i*_, *i* = 1, *m*), then the average quaternion can be computed ($\overline{q}$),

50 $$ \overline{q}=\frac{1}{m}\sum \limits_{i=1}^m{q}_i $$

With this approach after the averaging the quaternion is normalized to ensure the average is a unit quaternion. While such averaging is not statistically optimal it does provide superior results to the averaging of Cardan or Euler angles. An improved approach was presented by Markley et al. [13]. Once again given a sequence of *m* quaternions the matrix *M* is computed,

51 $$ M=\frac{1}{m}\sum \limits_{i=1}^m{q}_i{q}_i^T $$

The average quaternion is the eigenvector of matrix *M* corresponding to the maximum eigenvalue.

Rigid body averaging in biomechanics can occur in a number of circumstance, for example making multiple measurements so that averaging improves accuracy, or averaging repeat trials of the same task to produce a representative time series signal(s). In the former case the distribution of attitudes will be relatively small, and in the latter case potentially much larger. To illustrate averaging of rigid body attitudes, 1000 criterion attitude matrices were generated via exploiting a random number generator. For each criterion attitude matrix 10 noisy versions of the matrix were generated. The noisy matrices were generated based on the error model of Woltring et al. [27] where errors (Δφ) are multiplicative with an isotropic distribution. The noisy attitude matrix ($\hat{R}$) is,

52 $$ \hat{R}=\left(I+A\left(\Delta \upvarphi \right)\right)\ R $$

Where *I* is the identity matrix, and *A*(Δ*φ*) is a skew-symmetric matrix,

53 $$ A\left(\Delta \varphi \right)=\left[\begin{array}{ccc}0& -\Delta {\varphi}_z& \Delta {\varphi}_y\\ {}\Delta {\varphi}_z& 0& -\Delta {\varphi}_x\\ {}-\Delta {\varphi}_y& {\Delta \varphi}_x& 0\end{array}\right] $$

The error vector Δ*φ* refers to small rotational errors about the reference frame affixed to the body of interest. Three noisy conditions were examined, one with a noise standard deviation of 0.035° (e.g., [11]) to reflect errors which might occur in Roentgen stereo-photogrammetry, one with 2° to reflect the spread of performances which may occur if a subject performs the same task multiple times (e.g., [25]), and an extreme condition of 10°. For each of the 10 noisy attitude matrices the average rigid body attitude was determined by 1) computing the average of the Cardan angles determined from the noisy matrices, 2) computing the average of the quaternions determined from the noisy matrices, and 3) computing an average quaternion as the eigenvector of matrix *M* corresponding to the maximum eigenvalue. To assess the error in computing the average attitude the product of the attitude matrix estimate of the average and transpose of the criterion were computed,

54 $$ {R}_{err}={R}_{Est}{R}_{Criterion}^T $$

where *R*_*Criterion*_ is the criterion attitude matrix, and *R*_*Est*_ the estimated average attitude matrix. The error matrix (*R*_*err*_) can be quantified as the error angle (*θ*) which can be computed from,

55 $$ \theta ={\mathit{\cos}}^{-1}\left(\frac{trace\left({R}_{err}\right)-1}{2}\right) $$

If the estimated average attitude matrix exactly equals the criterion matrix, then the error matrix would be the identity matrix, giving an error angle of zero. This error angle reflects the angle through which the rigid body attitude defined by the estimated average attitude matrix must be rotated so that it corresponds with the attitude defined by the criterion attitude matrix (Chasles Theorem).

When the noise level is low all three methods produce the same performance (Table 2), this is to be expected as taking the average of a set of angles takes the chord to the surface of a sphere which for small noise levels is a reasonable approximation to the surface. With increasing noise level, the taking the average of a set of angles introduces larger errors than the other two approaches. The method of Markley et al. [13] is superior to the simple averaging of quaternions, and subsequent normalization, but the latter approach gives a reasonable approximation if speed of processing is important.

**Table 2:** The error angle corresponding to the estimation of rigid body attitude from multiple rigid body attitude measurements. Three methods are compared: the average of Cardan angles, the average and subsequent normalization of quaternions, and using the method of Markley et al. [13] for processing a set of quaternions

Method	Noise Standard Deviation		
	0.035	2	10
Average of Cardan Angles	0.009	0.90	4.23
Average of Quaternions	0.009	0.51	2.76
Markley et al. Quaternion Method	0.009	0.51	2.74

## Interpolating three-dimensional orientations

The three-dimensional attitude of a rigid body can be determined using a variety of methods, including image-based motion analysis or the use of an inertial measurement unit. Given these data there are a number of reasons why it might be interpolated. For example, when trying to temporally align signals collected at different rates, or to increase the temporal density of collected data. The increase of the temporal density of sampled data is appropriate if data collection has observed Shannon’s sampling theorem [17].

Vectors are relatively easily interpolated as vectors exist in linear space. In contrast quaternions exist in a curved space as each quaternion corresponds to a point on a unit hypersphere, therefore appropriate interpolation between pairs of quaternions must allow for the shape of the hypersphere surface. An appropriate approach to interpolating quaternions will ensure a consistent angular velocity between a pair of quaternions. The procedure typically used for quaternion interpolation is called **Slerp**, a name which derived from **S**pherical **l**inear int**erp**olation [20]. The Slerp formula for interpolating between two quaternions *q*_*1*_ and *q*_*2*_ is,

56 $$ q=\frac{\sin \left(\left(1-f\right)\theta \right)}{\sin \theta }{q}_1+\frac{\sin \left( f\theta \right)}{\sin \theta }{q}_2 $$

Where *θ* is the angle between the two quaternions (which can be computed from their dot product), and *f* is the fraction of interval between the two quaternions for which a quaternion is to be estimated (0 < *f* < 1).

**Fig. 4:**
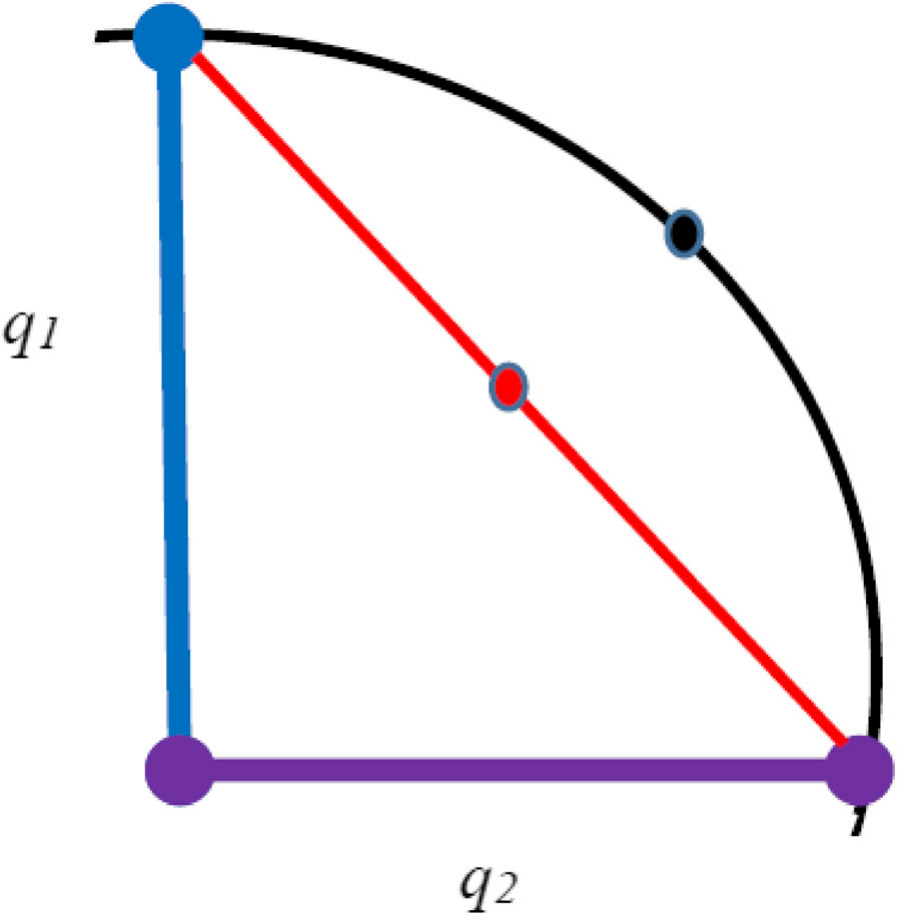
Spherical interpolation between the quaternions, *q*_*1*_ and *q*_*2*_. If simple linear interpolation was used then the interpolated point would be based on a line passing through the hypershere (the red colored chord). Using spherical interpolation, the predicted point is on the surface of the hypersphere

The errors which arise if rigid body attitude data is not appropriately interpolated parallel those which occur if these data are not appropriately averaged, with the errors arising being larger the greater the time interval over which interpolation is to be performed, as greater time is likely associated with greater motion. If linear interpolation is used the surface of the hypersphere is approximated by a chord (Fig. 4). Slerp ensures that interpolated points lie on the surface of the hypersphere.

## Three-dimensional orientations derivatives

Angular velocity is the rate of change of the orientation of one reference frame with respect to another, therefore the angular velocities cannot simply be computed from the differentiation of the orientation angles. Angular velocities can be computed from Poisson’s equation [26], for example given the attitude matrix (*R*) at a given time instant,

57 $$ A\left\{\omega \right\}=\dot{R}{R}^T $$

(Where) $$ A\left\{\omega \right\}=\left[\begin{array}{ccc}0& -{\omega}_Z& {\omega}_Y\\ {}{\omega}_Z& 0& -{\omega}_x\\ {}-{\omega}_Y& {\omega}_X& 0\end{array}\right] $$

The computation of the angular velocities from quaternions is straightforward,

58 $$ \left[\begin{array}{c}{\omega}_X\\ {}{\omega}_Y\\ {}{\omega}_Z\end{array}\right]=\left[\begin{array}{c}-{q}_1\\ {}-{q}_2\\ {}-{q}_3\end{array}\begin{array}{c}-{q}_0\\ {}{q}_3\\ {}-{q}_2\end{array}\begin{array}{c}-{q}_3\\ {}{q}_0\\ {}{q}_1\end{array}\begin{array}{c}{q}_2\\ {}-{q}_1\\ {}{q}_0\end{array}\right]\left[\begin{array}{c}{q}_0.\\ {}{q}_1.\\ {}{q}_2.\\ {}{q}_3.\end{array}\right] $$

## Conclusion

There is an efficiency to using quaternions. Compared with other approaches (e.g., Euler angles, Cardan angles), the quaternion does not suffer from singularities when defining rigid body orientation, and therefore avoids the gimbal lock. The quaternion represents the direction cosine matrix as a homogenous quadratic function of the components of the quaternion, unlike other approaches it does not require trigonometric or other transcendental function evaluations. It is also efficient for combining rotations, averaging rotations, interpolating rigid body orientations, and the computation of derivatives.

## Data Availability

Not applicable.

## Abbreviations

*e*: The rotation axis defined by a quaternion
*i, j, k*: Imaginary components of a quaternion where *i*^2^ = *j*^2^ = *k*^2^ = *ijk* = − 1,
*u*: The amount of translation along a helical axis
Ω: The angle of the rotation caused by a quaternion
α*,* β ,γ: Set of Euler or Cardan angles
*R*: *3* x *3 a*ttitude matrix
*SO*(3): The special-orthogonal group of order three
*U*, *D*, *V*: Components of the singular value decomposition of a matrix, where *U* is square orthogonal matrix, *D* is a diagonal matrix whose elements are non-negative real values (the singular values), and *V* is a *square* orthogonal matrix
*v*: is a *3* x *1* vector describing the translation from one pose to the another
*q*: A quaternion where *q* = *q*_0_ + *q*_1_*i* + *q*_2_*j* + *q*_3_*k*; *q*_0_, *q*_1_, *q*_2_, and *q*_3_ - real, components of a quaternion
*s*: The location of a point on a helical axis
